# Lithium and Chlorine-Rich Preparation of Mechanochemically Activated Antiperovskite Composites for Solid-State Batteries

**DOI:** 10.3389/fchem.2020.562549

**Published:** 2020-09-29

**Authors:** Yi Yang, Joah Han, Michael DeVita, Stephanie S. Lee, Jae Chul Kim

**Affiliations:** Department of Chemical Engineering and Materials Science, Stevens Institute of Technology, Hoboken, NJ, United States

**Keywords:** solid-state batteries, solid electrolytes, anti-perovskite, Li_3_OCl, passivation layer, ion-exchange

## Abstract

Assembling all-solid-state batteries presents a unique challenge due to chemical and electrochemical complexities of interfaces between a solid electrolyte and electrodes. While the interface stability is dictated by thermodynamics, making use of passivation materials often delays interfacial degradation and extends the cycle life of all-solid cells. In this work, we investigated antiperovskite lithium oxychloride, Li_3_OCl, as a promising passivation material that can engineer the properties of solid electrolyte-Li metal interfaces. Our experiment to obtain stoichiometric Li_3_OCl focuses on how the starting ratios of lithium and chlorine and mechanochemical activation affect the phase stability. For substantial LiCl excess conditions, the antiperovskite phase was found to form by simple melt-quenching and subsequent high-energy ball-milling. Li_3_OCl prepared with 100% excess LiCl exhibits ionic conductivity of 3.2 × 10^−5^ S cm^−1^ at room temperature, as well as cathodic stability against Li metal upon the extended number of cycling. With a conductivity comparable to other passivation layers, and stable interface properties, our Li_3_OCl/LiCl composite has the potential to stably passivate the solid-solid interfaces in all-solid-state batteries.

## Introduction

Technical advances in nonflammable solid electrolytes that can replace carbonate-based flammable liquid electrolytes of Li-ion batteries show promise toward the development of next-generation, ultimately safe solid-state batteries (Bachman et al., 2016; Manthiram et al., 2017). This all-solid system can also improve the cell packing efficiency and enable the use of lithium metal anodes to make batteries smaller, lighter, and longer-lasting (Famprikis et al., 2019). To date, a wide variety of promising solid electrolyte materials have been proposed, and thiophosphate-based materials in particular have received substantial interest due to high conductivity and robust mechanical properties (Lee et al., 2019). However, almost all thiophosphate electrolytes are stable only in a narrow voltage window and likely decompose if combined with the Li metal anode, as stated by thermodynamics (Richards et al., 2016; Zhu et al., 2016). Their decomposition products often include metallic phases that induce the continuous growth of a decomposition layer (Xiao et al., 2020). Indeed, continuous formation of Li_2_S, Li_3_P, and Li_15_Ge_4_ alloy (or Ge metal) is observed at the interface between Li_10_GeP_2_S_12_ and Li metal (Wenzel et al., 2016). Alternatively, the solid electrolyte-Li metal anode interface can be kinetically stabilized if the decomposition reaction leads to electronically insulating phases (Xiao et al., 2020). For Li_7_P_3_S_11_, decomposed Li_2_S and Li_3_P intrinsically passivate the Li_7_P_3_S_11_-Li metal interface with a self-limited thickness, affording sustained electrolyte function at the expense of cell polarization (Wenzel et al., 2016, 2018). Understanding the nature and evolution of decomposition products is thus critical to governing interfacial properties and developing the all-solid cell.

The compatibility between solid electrolytes and Li metal can be engineered by using extrinsic passivation materials that can decrease the electrochemical potential gradient across the interface (Zhu et al., 2015, 2016; Richards et al., 2016), such as aluminate, phosphate, and nitride (Miara et al., 2015; Sang et al., 2018; Zhang et al., 2018). Here, we investigate the electrochemical properties of antiperovskite lithium oxychloride with the nominal target composition Li_3_OCl as a passivation material to protect the solid electrolyte-Li metal interface. This material consists of no reduceable cations other than Li, making it electrochemically stable against Li metal (Xiao et al., 2020). Computational predictions and experimental evidence show that Li_3_OCl indeed forms stable interfaces against Li (Lü et al., 2016; Richards et al., 2016). Li_3_OCl can be feasibly prepared by a simple melt-quench method at low temperatures (Zhao and Daemen, 2012). However, recent studies showed that the Li conductivity and activation barrier of Li_3_OCl vary substantially with preparation methods, starting compositions, and sample morphologies, all of which likely affect the phase stability, and thus the mode of Li migration (Zhao and Daemen, 2012; Braga et al., 2014; Lü et al., 2014, 2016). The fundamental reasons that cause this discrepancy are unclear. In this work, to understand how starting compositions affect the lithium and chlorine stoichiometry of the antiperovskite and the associated Li conductivity, we prepared Li_3_OCl with various precursor ratios. Our results indicate that dry high-energy ball-milling of a substantial lithium and chlorine excess composition is critical to stabilize antiperovskite Li_3_OCl. Impedance spectroscopy and stability testing revealed that this phase exhibits reasonable Li conductivity, 3.21 × 10^−5^ S/cm, and is stable against Li metal.

## Materials and Methods

LiOH (98%, Sigma Aldrich) and LiCl (99%, Sigma Aldrich) with molar ratios of 2:*x* where *x* = 0.5, 1, 1.5, 2, and 2.5 were manually mixed in a nitrogen-filled glovebox. Note that stoichiometric *x* for Li_3_OCl is 1, with H_2_O evaporation (2LiOH + 2LiCl → Li_3_OCl + H_2_O). The mixtures were heat-treated at 350°C for 6 h and quenched to room temperature in ambient air. As they melt completely at 350°C, bead-like solids were formed upon quenching. The quenched samples were transferred immediately to an argon-filled glovebox to avoid air exposure. In the glovebox, high-energy ball-milling (Spex SamplePrep 8000M) was performed using zirconia grinding media in a zirconia container for 30 min. The crystal structure and phase compositions of the as-quenched and as-ball-milled materials were identified by X-ray diffraction (XRD, Bruker D8 Discover) with Cu Kα radiation. To prevent air-exposure, the XRD samples were sealed using a polyimide film (Kapton, DuPont). Microstructural and elemental analyses were performed on a scanning electron microscope (SEM, Zeiss Auriga).

To estimate the Li conductivity of Li_3_OCl samples, ball-milled powder specimens were uniaxially pressed to form disc-shaped pellets (10 mm diameter and ~0.3 mm thickness) in the glovebox, and silver paste was applied on both sides of the pellet as blocking electrodes. Electrochemical impedance spectroscopy (EIS) was carried out in a frequency range of 7 MHz−100 mHz with a 10 mV AC amplitude using a BioLogic SP-300 potentiostat/frequency response analyzer. Obtained EIS data were fitted using ZView software. For a variable temperature EIS test, a dedicated thermoelectric temperature chamber (BioLogic ITS) and a holder (BioLogic CESH) were used. A symmetric cell with Li/Li_3_OCl/Li configuration was built to assess interface stability against Li metal using a custom-made polytetrafluoroethylene Swagelok union with spring-loaded stainless-steel current collectors. A galvanostatic mode with current densities of 0.035 and 0.070 mA/cm^2^ was used to cycle the cell. The current polarity was switched every 30 min.

## Results

**Figure 1:**
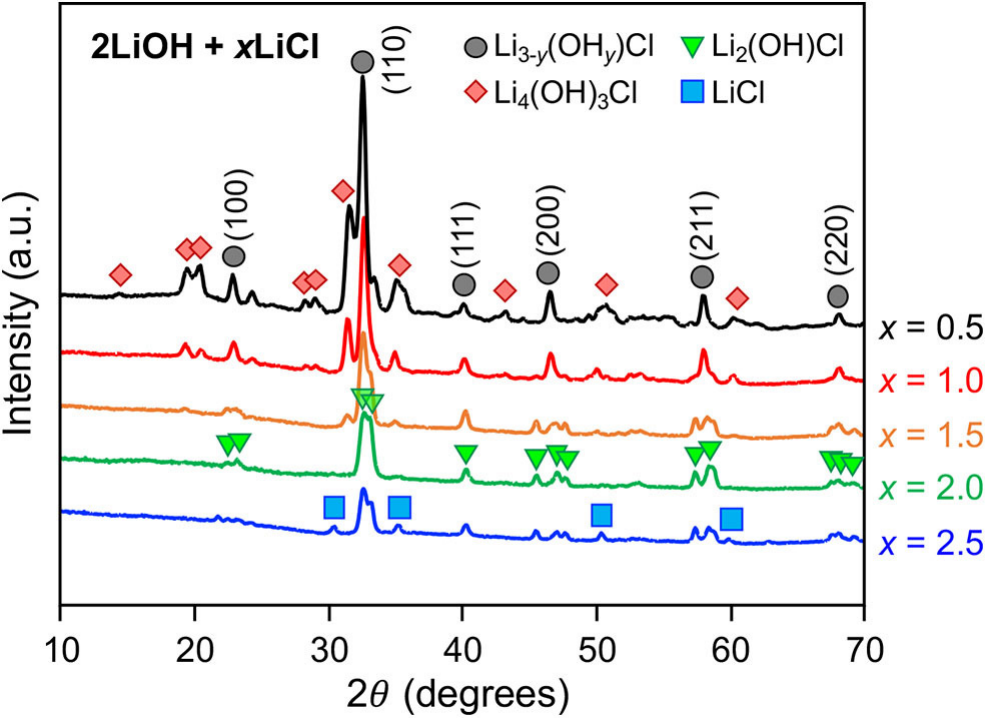
XRD patterns of as-quenched 2LiOH-*x*LiCl samples with *x* = 0.5, 1, 1.5, 2, and 2.5.

Figure 1 shows XRD patterns of as-quenched 2LiOH-*x*LiCl (*x* = 0.5, 1, 1.5, 2, 2.5) measured in transmission mode. For *x* = 0.5 and 1, observed major peaks display cubic symmetry in the *Pm3m* space group, suggesting the formation of an antiperovskite Li_3_OCl phase. However, hydrated variants of Li_3_OCl can also crystallize in the cubic symmetry with similar lattice parameters (Schwering et al., 2003; Hanghofer et al., 2018). As their XRD patterns are indistinguishable, we denote this cubic phase as Li_3−y_(OH_*y*_)Cl (0 ≤ *y* < 1). Peaks for a secondary phase likely corresponds to monoclinic Li_4_(OH)_3_Cl, as identified by Rettenwander and coworkers (Hanghofer et al., 2018), though other lithium hydroxychloride forms may coexist. As *x* increases from 0.5 to 1, the Li_4_(OH)_3_Cl-to-Li_3−y_(OH_*y*_)Cl peak intensity ratio decreases, consistent with the hydroxide/chlorine stoichiometry of the nominal compositions. For a higher LiCl content of *x* = 1.5, the peak intensity for Li_4_(OH)_3_Cl decreases substantially. Also, we observed peak splitting of the antiperovskite phase, indicating symmetry reduction, which matches the structure of orthorhombic Li_2_(OH)Cl (Li et al., 2016). Li_2_(OH)Cl has higher relative chlorine content compared to Li_4_(OH)_3_Cl, consistent with our observation that its formation is favored under excess LiCl content. By further LiCl addition, the sample at *x* = 2 primarily consists of Li_2_(OH)Cl. For this composition, *x* = 2 is the stoichiometric value assuming no water evaporation, with 2LiOH + 2LiCl → 2Li_2_(OH)Cl. The peak intensity of Li_2_(OH)Cl decreases at *x* = 2.5, and peaks for LiCl emerge.

**Figure 2:**
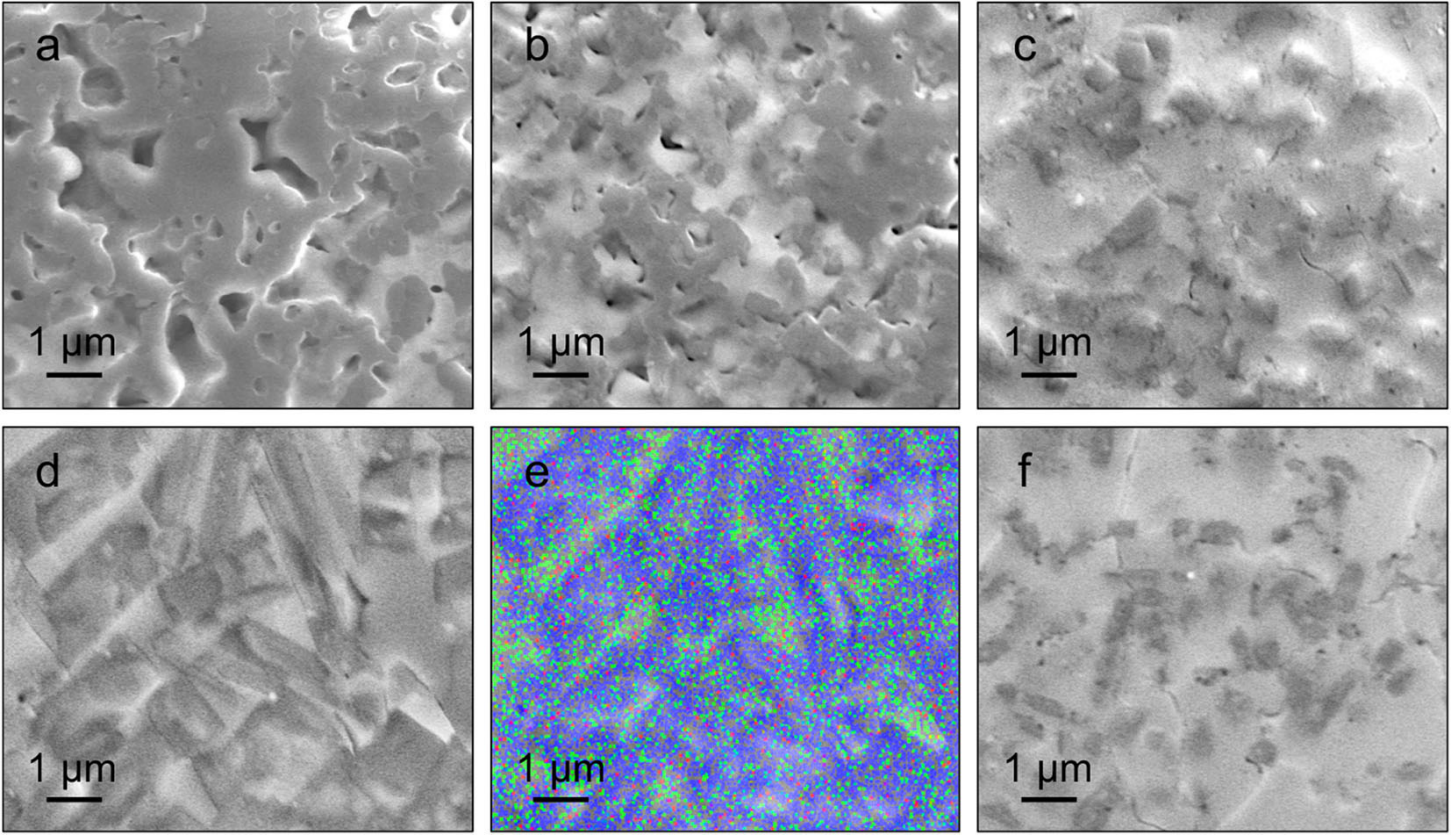
SEM images of 2LiOH-*x*LiCl where *x* = **(a)** 0.5, **(b)** 1, **(c)** 1.5, **(d,e)** 2, and **(f)** 2.5 samples. The EDS result for *x* = 2 is shown in **(e)** (Blue: oxygen, Green: chlorine).

Figure 2 shows the cross-sectional SEM images obtained from as-quenched 2LiOH-*x*LiCl solids. The microstructure of the sample prepared with an LiCl deficiency, *x* = 0.5, in Figure 2a is unstructured, which is common in glass-ceramics made by melt-quenching (Deubener et al., 2018). As *x* increases to 1 and 1.5, aggregated square/rectangular precipitates embedded within an amorphous-like matrix appear, as shown in Figures 2b,c. XRD spectra presented in Figure 1 suggest that these precipitates comprise Li_3−y_(OH_*y*_)Cl and Li_3_OCl. In Figure 2d, precipitated domains have the most distinct rectangular morphology when *x* = 2. According to energy dispersive X-ray spectroscopy in Figure 2e, we found that oxygen and chlorine distribution across the *x* = 2 sample is inhomogeneous. The precipitates contain both oxygen and chlorine, while the matrix contains mostly chlorine, suggesting that the matrix likely consists of amorphized and/or hydrated LiCl undetectable by XRD. In Figure 2f, the crystals are covered excessively by the matrix due to the substantial amount of LiCl addition, *x* = 2.5.

**Figure 3:**
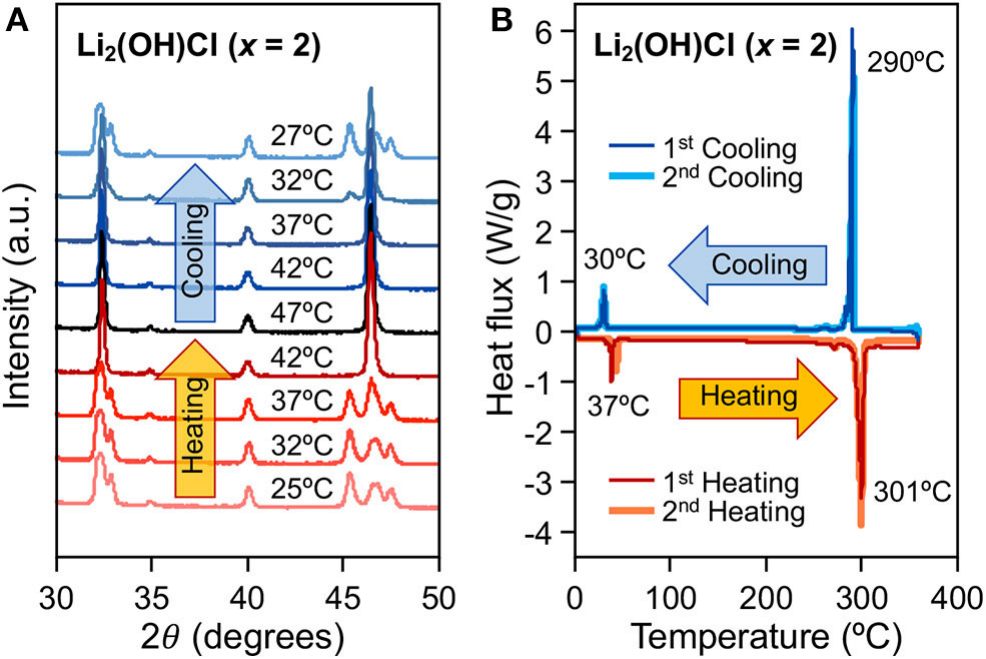
**(A)** *in situ* XRD and **(B)** differential scanning calorimetry of as-quenched Li_2_(OH)Cl.

We further analyzed the phase stability of Li_2_(OH)Cl (*x* = 2 in 2LiOH-*x*LiOH) under ambient and elevated temperatures using *in situ* XRD. In Figure 3A, split peaks at 32 and 46° 2θ of the as-quenched orthorhombic structure merge into single peaks upon heating from 37 to 42°C. This indicates an orthorhombic-to-cubic solid-state phase transition, which is reversed upon cooling from 32 to 27°C. Differential scanning calorimetry also confirms the existence of a reversible phase transition during two repetitive heating and cooling cycles. As shown in Figure 3B, small endothermic and exothermic peaks at 37 and 30°C correspond to the orthorhombic-to-cubic and cubic-to-orthorhombic phase transitions, respectively. Note that large peaks observed at 301 and 290°C are due to the melting and recrystallizing of Li_2_(OH)Cl. Our observation of a reversible solid-state transition for Li_2_(OH)Cl around 35°C agrees with previous reports in the literature (Schwering et al., 2003; Hanghofer et al., 2018; Song et al., 2020).

**Figure 4:**
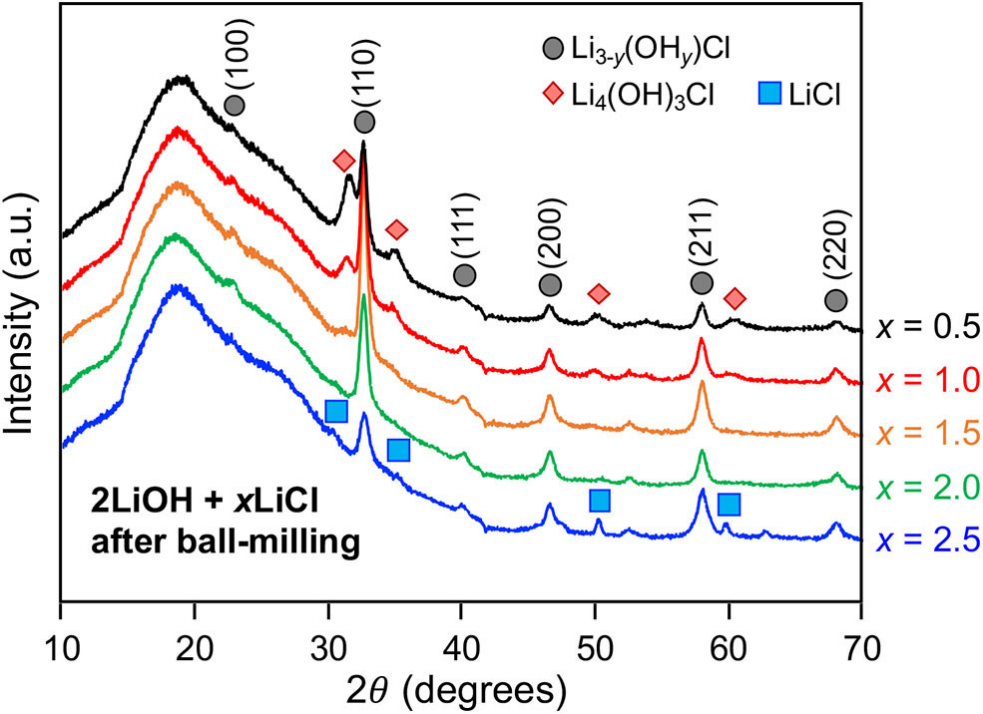
XRD patterns of as-ball-milled 2LiOH-*x*LiCl (*x* = 0.5, 1, 1.5, 2, 2.5).

Mechanochemically, high-energy ball-milling often triggers phase transitions that do not require long-range atomic diffusion (Shi et al., 2018). We investigated how the high-energy ball-milling process affects the composition of the antiperovskite and secondary phases. The quenched 2LiOH-*x*LiCl (*x* = 0.5, 1, 1.5, 2, 2.5) solids were subsequently ball-milled in the argon-filled glovebox. Figure 4 shows XRD patterns of as-ball-milled powder samples. A broad hump around 18° 2θ belongs to a polyimide film that was used to seal the specimen. For *x* = 0.5 and 1, the XRD patterns match those of as-quenched samples displayed in Figure 1, indicating that the overall phase composition, Li_3−y_(OH_*y*_)Cl and Li_4_(OH)_3_Cl, before and after high-energy ball-milling remains unchanged. Peak broadening upon ball-miling is likely due to particle size reduction and/or the formation of crystallographic defects. Interestingly, ball-milling changes the phase composition substantially for 2LiOH-*x*LiCl with higher LiCl content. For *x* = 1.5, 2, and 2.5, the peaks associated with orthorhombic Li_2_(OH)Cl before ball-milling are replaced with peaks that can be indexed to the antiperovskite structure. Note that a small amount of monoclinic Li_4_(OH)_3_Cl still exists for *x* = 1.5. For the *x* = 2 sample, only diffraction peaks consistent with the antiperovskite phase were observed. The intensities of the peaks associated with the antiperovskite phase decreased for the sample with *x* = 2.5, and peaks for LiCl that were present before ball-milling remained.

It is worthwhile to discuss the formation mechanism of the antiperovskite phase, especially for lithium and chlorine-rich 2LiOH-*x*LiCl (*x* > 1) observed in Figure 4. Experimentally, the stability of antiperovskite Li_3_OCl is moisture-sensitive, in which hydration leads to phase transformation/degradation under ambient temperature and pressure (Hanghofer et al., 2018). This also implies that the stoichiometric mixing of 2LiOH and LiCl unlikely leads to pure antiperovskite formation due to hydroxide formation in moist air. Consistently, we observed the monoclinic Li_4_(OH)_3_Cl phase if near-stoichiometric values of *x* in mixing 2LiOH and *x*LiCl are used or Li_2_(OH)Cl if substantial excess LiCl is involved. The pure antiperovskite phase can be obtained by transforming orthorhombic Li_2_(OH)Cl via high-energy ball-milling. This result is very interesting because the cubic form of Li_2_(OH)Cl is unstable at room temperature, as demonstrated in Figure 3. At room temperature, stable cubic antiperovskite compositions reported in literature are Li_1.16_(OH_1.84_)Cl, Li_2.17_(OH_0.83_)Cl, and Li_3_OCl (Schwering et al., 2003; Zhao and Daemen, 2012). Given the excess amount of lithium and chlorine, we consider that the obtained antiperovskite in Figure 4 is Li-rich Li_3−y_(OH_*y*_)Cl (0 ≤ *y* < 1). The orthorhombic-to-cubic transition can be plausibly explained by a dynamic ion exchange reaction between protons in Li_2_(OH)Cl and lithium ions in surrounding residual LiCl facilitated by high-energy ball-milling. As LiCl also affords a wide range of hydration (Ruiz et al., 2014), which may further facilitate the H^+^/Li^+^ ion-exchange reaction, the resulting antiperovskite phase can even be close to stoichiometric Li_3_OCl antiperovskite. Indeed, the estimated lattice parameter from the (110) peak position at 32° is *a* = 3.916 Å, in a good agreement obtained from theoretical investigation of Li_3_OCl (Deng et al., 2015; Lü et al., 2016; Dawson et al., 2018b). Thus, we hereafter denote the antiperovskite phase obtained by high-energy ball-milling for *x* =2 as Li_3_OCl. Note that the sample should be a composite of Li_3_OCl and hydrated LiCl. Ball-milling does not affect the phase composition at *x* = 0.5 and 1 due to limited availability of LiCl. Also, Li_4_(OH)_3_Cl tends to be more stable than Li_2_(OH)Cl (Hanghofer et al., 2018), which may sustain the composition better against the ion-exchange reaction.

**Figure 5:**
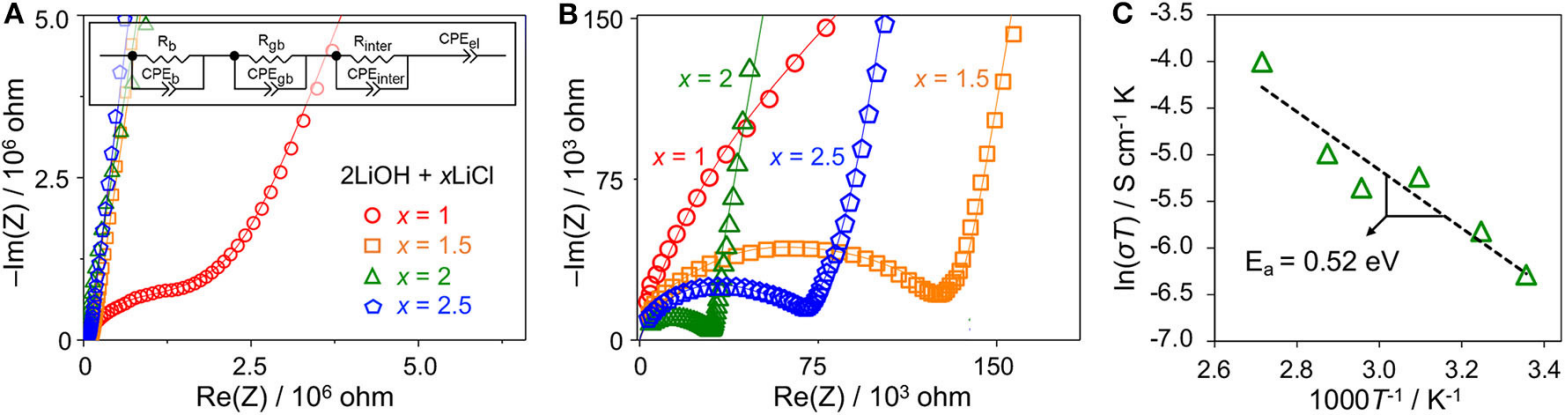
**(A,B)** Nyquist plots of high-energy ball-milled 2LiOH-*x*LiCl (*x* = 1, 1.5, 2, 2.5) in two different scales. The inset illustrates an equivalent circuit used to fit the EIS data. **(C)** The Arrhenius plot of the Li_3_OCl composite sample (*x* = 2).

We evaluated the Li conductivity of high-energy ball-milled 2LiOH-*x*LiCl (*x* = 1, 1.5, 2, 2.5) by EIS. Figures 5A,B are their Nyquist plots for two different scales. Depressed semicircles are observed for all samples in the high frequency range followed by a straight line in the low frequency region. The radius of the semicircle decreases as *x* increases from 0.5 to 2. Compared with the smallest semicircle observed for the antiperovskite composite at *x* = 2, the radius increases by a factor of two at *x* = 2.5, as shown in Figure 5B. To obtain Li conductivity, we fit our EIS data using an equivalent circuit (the Figure 5A inset) that consists of three parallel segments of resistor and constant phase element (CPE), each of which is connected in series to represent bulk (b), grain boundary (gb), and interphase (inter) impedances, and a single serial CPE to fit a blocking electrode. To calculate total Li conductivity (σ), we have used the total resistance of the three segments combined (i.e., *R* = *R*_*b*_+*R*_*gb*_+*R*_*int*_) and calculated σ = *R*^−1^(*l*/*A*) where *l* and A stand for sample thickness and diameter, respectively. The total conductivity increases as *x* increases from 1 to 2: 1.15 × 10^−6^ S/cm for *x* = 1, 1.76 × 10^−5^ S/cm for *x* = 1.5, and 3.21 × 10^−5^ S/cm for *x* = 2. The conductivity decreases to 1.24 × 10^−5^ S/cm for *x* = 2.5. These results are consistent qualitatively with the relative antiperovskite composition identified in Figure 4. The larger antiperovskite content leads to higher Li conductivity due to low ionic conductivities of Li_4_(OH)_3_Cl and hydrated LiCl (Schwering et al., 2003; Hanghofer et al., 2018). Note that the computed bulk and grain boundary Li conductivities of Li_3_OCl are on the order of 10^−5^ and 10^−6^ S/cm (Deng et al., 2015; Dawson et al., 2018b). Figure 5C plots the Li conductivities of the Li_3_OCl (*x* = 2) composite as a function of temperature. An activation barrier *E*_*a*_ of Li diffusion across the sample was estimated using the Arrhenius-type equation σT = Aexp(−*E*_*a*_/*k*_*B*_*T*) where σ is total ionic conductivity measured by EIS, *A* is a conductivity pre-factor, *k*_*B*_ is the Boltzmann constant, and *T* is temperature. The activation barrier obtained from the slope is 0.52 eV, consistent with a computational result for polycrystalline Li_3_OCl (Dawson et al., 2018b). It should be noted that although we do not fully exclude a possibility of residual hydroxide in the material at *x* = 2, a proton contribution to the total ionic conductivity is unlikely due to localized H^+^ motion (Dawson et al., 2018a).

**Figure 6:**
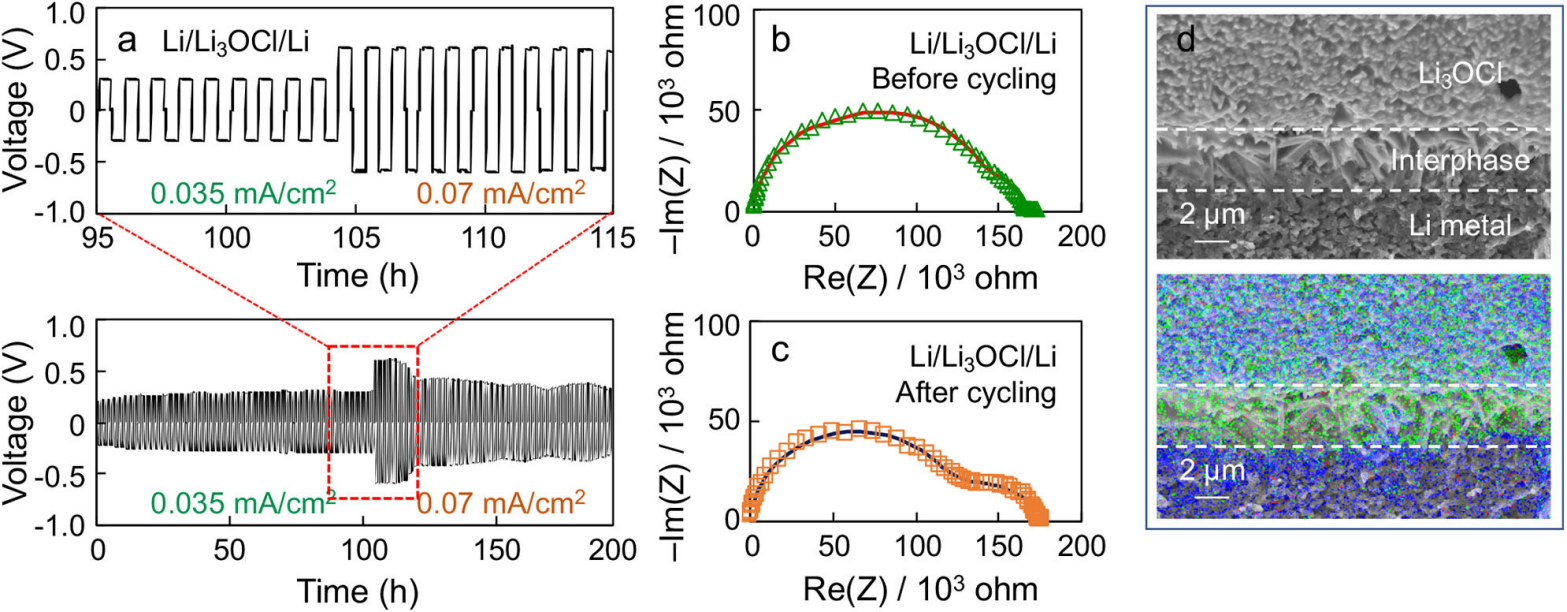
**(a)** Voltage polarization profile of a Li/Li_3_OCl/Li symmetric cell cycled at 0.035 and 0.07 mA/cm^2^ at room temperature. Nyquist plots of the Li symmetric cell **(b)** before and **(c)** after cycling. **(d)** Cross-sectional SEM image and EDS results of the cycled symmetrical cell at the Li_3_OCl-Li interface. (Blue: oxygen, Green: chlorine).

Figure 6a shows a representative voltage polarization profile of a Li symmetric cell made by the Li_3_OCl composite with 100% excess LiCl (*x* = 2) under two different current densities as a function of time. A stable voltage polarization profile is observed under the positive and negative current densities (0.035 mA/cm^2^), which is symmetric about 0 V. If the current density is doubled (0.07 mA/cm^2^), the polarization voltage is also doubled (i.e., ohmic behavior, while maintaining the stably alternating profile). Note that the cell polarization decreases after 120 h, indicative of Li penetration into the cold-pressed Li_3_OCl pellet. Figures 6b,c compare the EIS results of the symmetric cell before and after cycling. A low-frequency semicircle becomes evident upon cycling, indicative of interphase formation at the Li_3_OCl-Li metal interface. The EIS results were fitted using an equivalent circuit that has bulk, grain boundary, and interphase components. The interphase impedance increases from 28 to 61 kΩ upon cycling, suggesting the formation of surface phases, such as LiCl. Note that the sum of bulk and grain boundary contributions decreases from 138 to 115 kΩ. This result reflects the decrease in effective pellet thickness due to uneven Li plating. We hypothesize this interphase formation to be the result of small amounts of Li_3_OCl decomposition into LiCl and Li_2_O. Although expected to be electrochemically stable against Li metal due to the absence of reduceable elements other than Li, self-decomposition of metastable Li_3_OCl into Li_2_O and LiCl is possible according to computation (Emly et al., 2013). Figure 6d shows a cross-sectional SEM image and the corresponding EDS map of the Li_3_OCl-Li metal interface after cycling. It can be seen that a Cl-rich layer exists in between Li_3_OCl and Li metal, consistent with the EIS analysis and the computational result. Note that the Cl-rich layer may also originate from the excess amount of amorphized lithium chloride present in the sample. The oxygen signal on the Li metal side is likely due to the air-exposure during sample loading. We will scrutinize the interface structure using more advanced technique, such as cryogenic transmission electron microscopy, in future work. These results demonstrate the electrochemical compatibility of Li_3_OCl against Li, which satisfies the requirement to stably passivate solid electrolyte-Li metal anode interfaces.

## Conclusion

We investigate how stoichiometry affects the phase stability and Li conductivity of antiperovskite-type lithium-rich oxychloride, Li_3_OCl. The history of sample treatment leads to the substantial variation in the stoichiometry of Li_3_OCl and thus its Li conductivity. If prepared by a melt-quench method, we found that a substantial amount of excess LiCl leads to orthorhombic Li_2_(OH)Cl formation. An orthorhombic-to-cubic phase transition occurs upon high-energy ball-milling under inert atmosphere. Our results suggest that excess amounts of LiCl is the key to stabilize the antiperovskite Li_3_OCl structure during high-energy ball-milling, and solid-state H^+^/Li^+^ ion-exchange is proposed as a plausible stabilization mechanism. We obtained consistently high conductivity and good electrochemical stability against Li metal. A lithium and chlorine rich-Li_3_OCl composite in this work shows a reasonable room-temperature Li conductivity and good cathodic stability against Li metal. With these desirable electrochemical properties, Li_3_OCl can be a promising material to passivate solid-solid interfaces in all-solid-state Li batteries.

## Data Availability Statement

The raw data supporting the conclusions of this article will be made available by the authors, without undue reservation.

## Author Contributions

YY and MD performed the experiments. JH analyzed impedance results. SL and JK supervised the project. All authors contributed to the article and approved the submitted version.

## Conflict of Interest

The authors declare that the research was conducted in the absence of any commercial or financial relationships that could be construed as a potential conflict of interest.
